# Supplementary material for: Pten controls B‐cell responsiveness and germinal center reaction by regulating the expression of IgD BCR
Source: EMBO J. 2019 Apr 23;38(11):e100249. doi: 10.15252/embj.2018100249 (PMC6545559; doi:10.15252/embj.2018100249)
Supplement: Supplementary file 1 — Appendix [file EMBJ-38-e100249-s001.pdf]

# Appendix - table of contents

**Appendix Figure S1 |** Pten-deficient B cells are capable of acquiring an anergic phenotype (related to Fig. 2)

**Appendix FigureS2 |** Pten is important for development of Fo.B cells (related to Fig. 3)

**Appendix Figure S3 |** Investigating direct FoxO1 binding to Fo.B cell-specific genes (related to Fig. 4)

**Appendix Table S1:** P values (related to Fig. 2C)

**Appendix Table S2:** P values (related to Fig. 2D)

**Appendix Table S3:** P values (related to Fig. 2E)

**Appendix Table S4:** P values (related to Fig. 3D)

**Appendix Table S5:** P values (related to Fig. 6A)

**Appendix Table S6:** P values (related to Fig. 6C)

**Appendix Table S7:** P values (related to Fig. 7G)

**Appendix Table S8 |** Antibodies used in flow cytometric analyses (related to Experimental Procedures)

**Appendix Table S9 |** Antibodies used for immunohistochemistry (related to Experimental Procedures)

**Appendix Table S10 |** Primers used for analysis of *LC* gene recombination (related to Experimental Procedures)

**Appendix Table S11 |** TaqMan-probe mixes used for qPCR (related to Experimental Procedures)

**Chromatin Immunoprecipitation (ChIP)** (related to Experimental Procedures)

**Appendix Table S12 |** Primers used for qPCR amplification of sequences with potential FoxO1-binding sites in the *Zfp318* gene
(related to Experimental Procedures)

**Appendix Table S13 |** Primers used for qPCR amplification of sequences with potential FoxO1-binding sites in the *Ell2* gene
(related to Experimental Procedures)

**Appendix Table S14 |** Primers used for qPCR amplification of sequences with potential FoxO1-binding sites in the *Fcer2* gene
(related to Experimental Procedures)

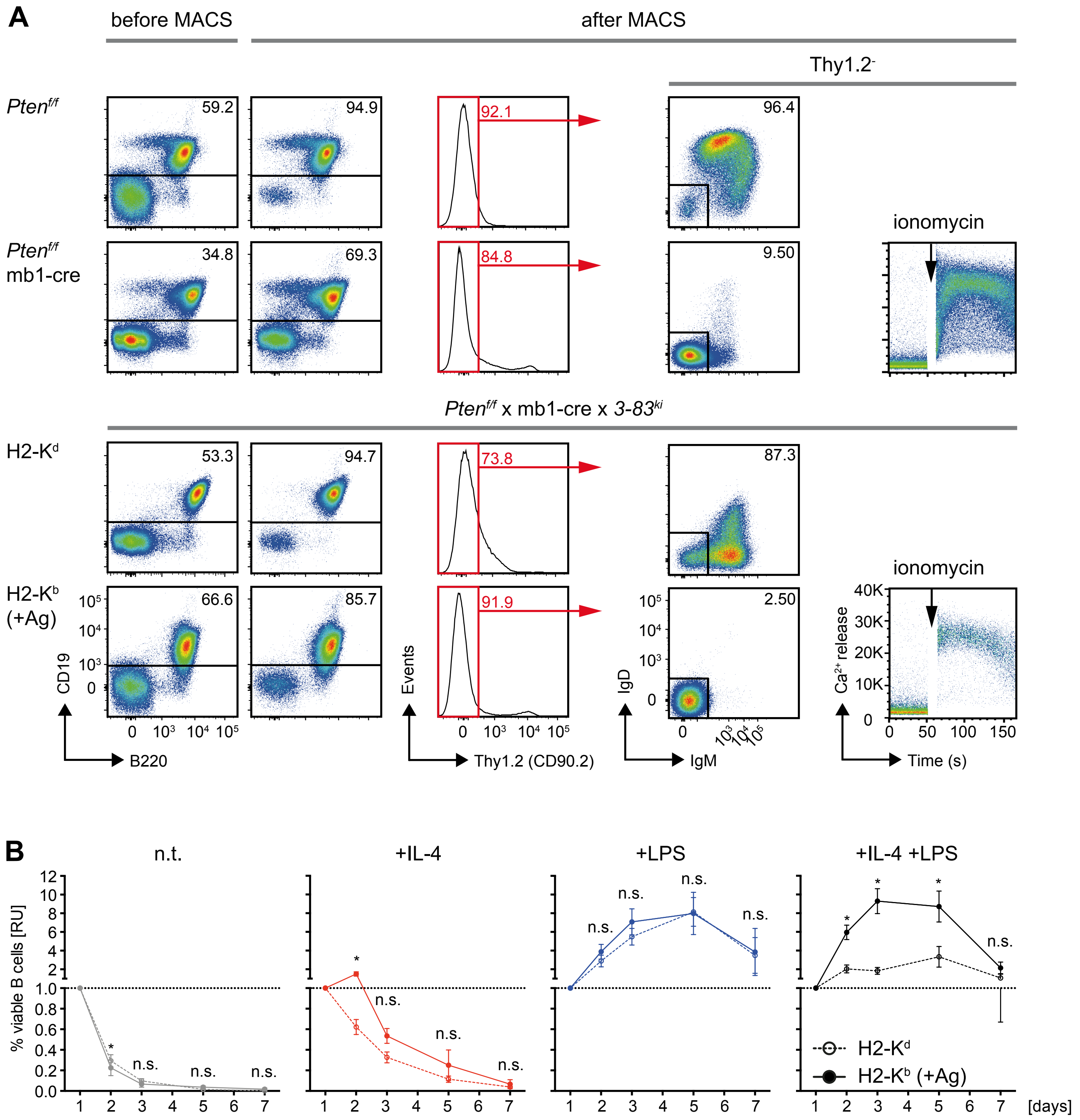


**Appendix Figure S1 | Pten-deficient B cells are capable of acquiring an anergic phenotype** (related to Fig. 2)

**A |** Mice of the indicated genotypes were sacrificed and mature splenic B cells were enriched by magnet-activated cell sorting (MACS). The purification efficiency was determined by flow cytometry (CD19/B220). IgM/IgD surface expression was analyzed in the Thy1.2$^-$ population, which was used for measurement of $Ca^{2+}$ mobilization in Fig. 2B. Mature splenic B cells that did not show $Ca^{2+}$ mobilization in Fig. 2B were stimulated with ionomycin to verify their capacity to induce $Ca^{2+}$ influx (right).

**B |** Splenic B cells from Pten$^{f/f}$ x mb1-cre x 3-83$^{ki}$ mice on the respective background (H2-K$^d$: w/o Ag, n = 4; H2-K$^b$: + Ag, n = 5) were purified and cultured for 7 days in presence of either IL-4 (10 ng/ml), LPS (2.5 µg/ml), the combination of both substances, or were left untreated (n.t. = non-treated). Percentages of viable B cells, determined at days 2, 3, 5 and 7 by flow cytometry, were normalized to those measured at day 1, (RU = relative units, mean ±SEM). Statistical significance was calculated by applying the Mann-Whitney U-test, n.s. = not significant, * $p \leq 0.05$.

# Appendix Figure S1

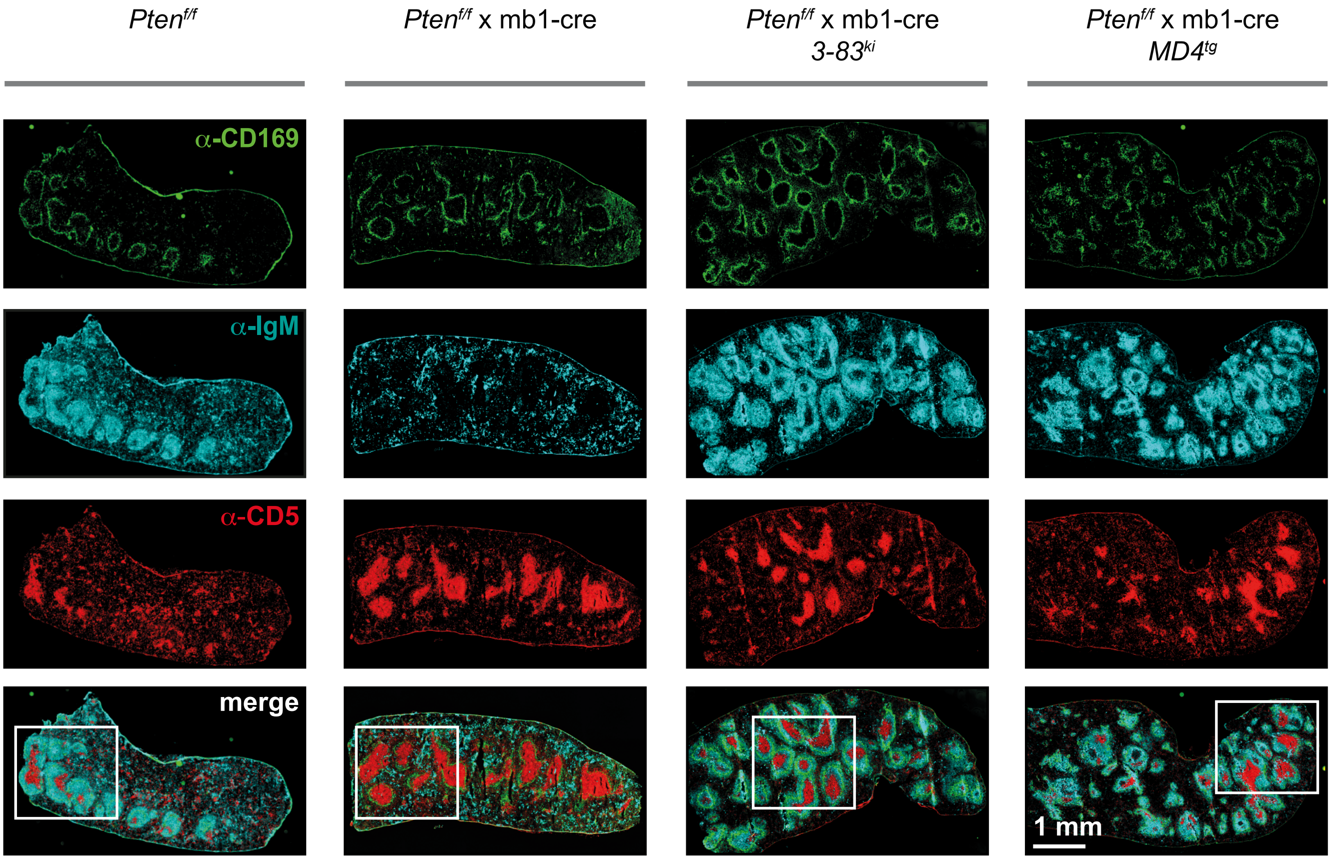


**Appendix Figure S2 | Pten is important for development of Fo.B cells** (related to Fig. 3)
Immunohistochemistry of sections from spleens of *Pten*$^{f/f}$, *Pten*$^{f/f}$x mb1-cre, *Pten*$^{f/f}$x mb1-cre x *3-83*$^{ki}$ and *Pten*$^{f/f}$ x mb1-cre x *MD4*$^{tg}$ mice for CD169 (green), CD5 (red) and IgM (cyan) at 10x magnification. Shown pictures are representative of 2-3 mice per genotype and display an overview of the whole section. Pictures shown in Fig. 3E represent enlarged views of the areas indicated by the white squares, respectively.

# Appendix Figure S2

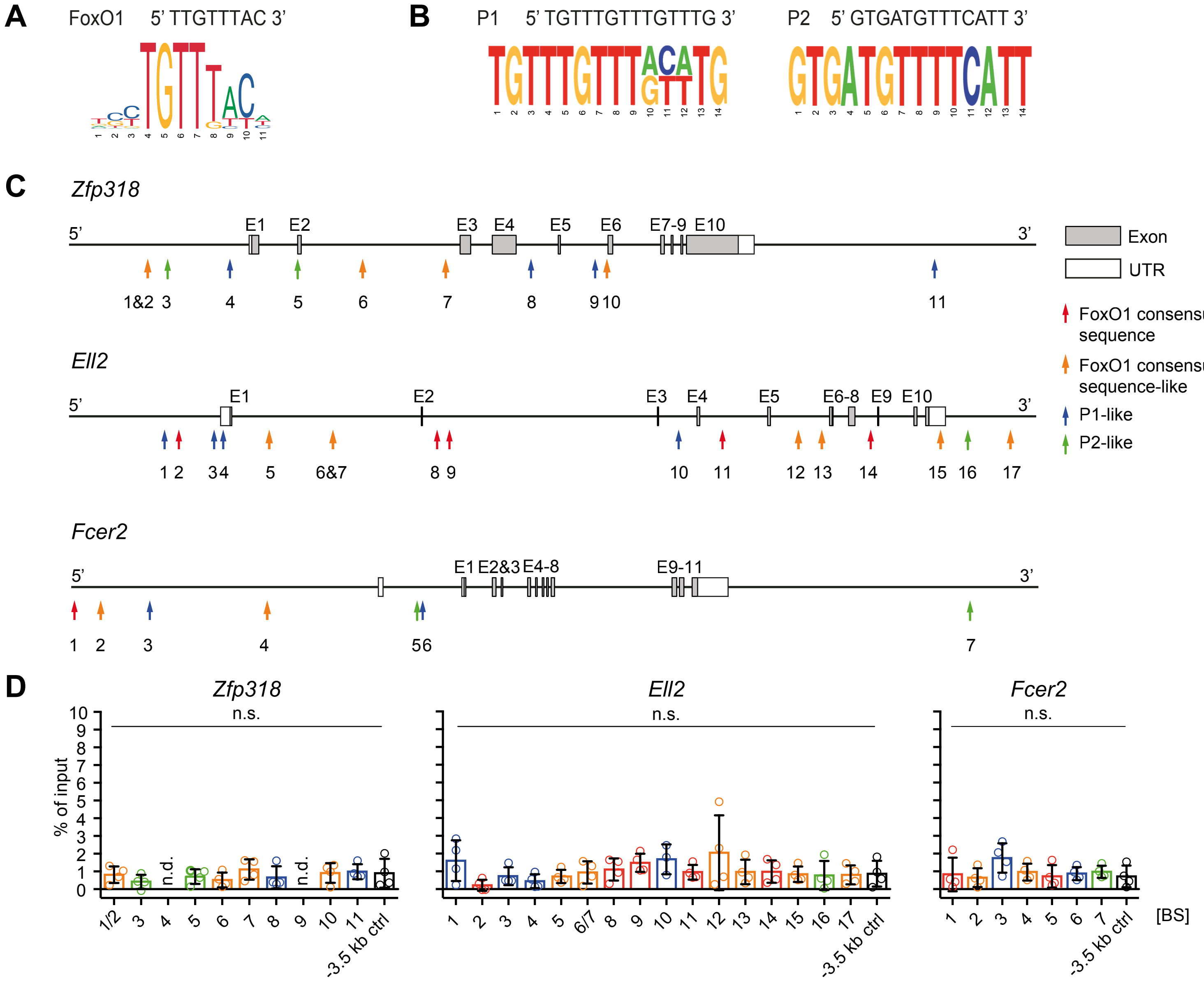


**Appendix Figure S3 | Investigating direct FoxO1-binding to Fo.B cell-specific genes** (related to Fig. 4)

**A |** Sequence of FoxO1-binding motif obtained from the JASPAR database.

**B |** Sequence of two FoxO1-binding motifs in the *Pax5* gene.

**C |** Schematic overview of the *Zfp318*, *Ell2* and *Fcer2* gene loci showing exons (gray boxes), untranslated regions (UTR, white boxes) and potential FoxO1-binding sites (arrows): 8/8 nucleotides matching FoxO1 consensus-sequence shown in Fig. EV3A (red), 7/8 nucleotides matching FoxO1 consensus-sequence (orange), sequences similar to P1 (blue) and P2 (green) shown in Fig. EV3B.

**D |** FoxO1 ChIP from mature B cells purified from spleens of WT mice (n = 4, except for Ell2-10 n = 3, mean ±SD). Sequences comprising the potential FoxO1-binding sites (BS) were amplified by quantitative PCR (for primer sequences see Appendix Tables S12 - 14). Amplification of a sequence containing no predicted FoxO1-binding site 3.5 kb downstream of the 3'-UTR in each gene was used as a negative control (-3.5 kb ctrl). Statistical significance was calculated by using the Kruskal-Wallis test between the data obtained for the individual binding sites and the -3.5 kb ctrl, n.d. = product not detectable on fragmented ChIP-enriched DNA, n.s. = not significant.

## Appendix Figure S3

## Supplemental information

### Appendix Table S1: related to Fig. 2C

| Kruskal-Wallis test: Dunn's multiple comparisons test | | Adjusted p value | |
|---|---|---|---|
| *Pten*$^{f/f}$ | *Pten*$^{f/f}$ x mb1-cre x *3-83*$^{ki}$ -Ag | > 0.9999 | n. s. |
| *Pten*$^{f/f}$ | *Pten*$^{f/f}$ x mb1-cre x *3-83*$^{ki}$ +Ag | < 0.0001 | **** |
| *Pten*$^{f/f}$ x mb1-cre | *Pten*$^{f/f}$ x mb1-cre x *3-83*$^{ki}$ -Ag | > 0.9999 | n. s. |
| *Pten*$^{f/f}$ x mb1-cre | *Pten*$^{f/f}$ x mb1-cre x *3-83*$^{ki}$ +Ag | < 0.0001 | **** |

### Appendix Table S2: related to Fig. 2D

| Kruskal-Wallis test: Dunn's multiple comparisons test | | Adjusted p value | |
|---|---|---|---|
| *Pten*$^{f/f}$ | *Pten*$^{f/f}$ x mb1-cre x *3-83*$^{ki}$ -Ag | 0.0129 | * |
| *Pten*$^{f/f}$ | *Pten*$^{f/f}$ x mb1-cre x *3-83*$^{ki}$ +Ag | 0.0288 | * |
| *Pten*$^{f/f}$ x mb1-cre | *Pten*$^{f/f}$ x mb1-cre x *3-83*$^{ki}$ -Ag | 0.0723 | n. s. |
| *Pten*$^{f/f}$ x mb1-cre | *Pten*$^{f/f}$ x mb1-cre x *3-83*$^{ki}$ +Ag | 0.0003 | *** |

### Appendix Table S3: related to Fig. 2E

| Kruskal-Wallis test: Dunn's multiple comparisons test | | Adjusted p value | |
|---|---|---|---|
| *Pten*$^{f/f}$ | *Pten*$^{f/f}$ x mb1-cre x *3-83*$^{ki}$ -Ag | 0.0720 | n. s. |
| *Pten*$^{f/f}$ | *Pten*$^{f/f}$ x mb1-cre x *3-83*$^{ki}$ +Ag | > 0.9999 | n. s. |
| *Pten*$^{f/f}$ x mb1-cre | *Pten*$^{f/f}$ x mb1-cre x *3-83*$^{ki}$ -Ag | 0.1240 | n. s. |
| *Pten*$^{f/f}$ x mb1-cre | *Pten*$^{f/f}$ x mb1-cre x *3-83*$^{ki}$ +Ag | < 0.0001 | **** |

### Appendix Table S4: related to Fig. 3D

| Kruskal-Wallis test: Dunn's multiple comparisons test | | Adjusted p value | |
|---|---|---|---|
| **5 mg/ml (row 1)** | | | |
| *Pten*$^{f/f}$ x *MD4*$^{tg}$ | *Pten*$^{f/f}$ x *MD4*$^{tg}$ x *ML5*$^{tg}$ | 0.0003 | *** |
| *Pten*$^{f/f}$ x mb1-cre x *MD4*$^{tg}$ | *Pten*$^{f/f}$ x mb1-cre x *MD4*$^{tg}$ x *ML5*$^{tg}$ | > 0.9999 | n. s. |
| **Dilution 1:3 (row 2)** | | | |
| *Pten*$^{f/f}$ x *MD4*$^{tg}$ | *Pten*$^{f/f}$ x *MD4*$^{tg}$ x *ML5*$^{tg}$ | 0.0003 | *** |
| *Pten*$^{f/f}$ x mb1-cre x *MD4*$^{tg}$ | *Pten*$^{f/f}$ x mb1-cre x *MD4*$^{tg}$ x *ML5*$^{tg}$ | > 0.9999 | n. s. |
| **Dilution 1:9 (row 3)** | | | |
| *Pten*$^{f/f}$ x *MD4*$^{tg}$ | *Pten*$^{f/f}$ x *MD4*$^{tg}$ x *ML5*$^{tg}$ | 0.0011 | ** |
| *Pten*$^{f/f}$ x mb1-cre x *MD4*$^{tg}$ | *Pten*$^{f/f}$ x mb1-cre x *MD4*$^{tg}$ x *ML5*$^{tg}$ | > 0.9999 | n. s. |
| **Dilution 1:27 (row 4)** | | | |
| *Pten*$^{f/f}$ x *MD4*$^{tg}$ | *Pten*$^{f/f}$ x *MD4*$^{tg}$ x *ML5*$^{tg}$ | 0.0017 | ** |
| *Pten*$^{f/f}$ x mb1-cre x *MD4*$^{tg}$ | *Pten*$^{f/f}$ x mb1-cre x *MD4*$^{tg}$ x *ML5*$^{tg}$ | > 0.9999 | n. s. |
| **Dilution 1:81 (row 5)** | | | |
| *Pten*$^{f/f}$ x *MD4*$^{tg}$ | *Pten*$^{f/f}$ x *MD4*$^{tg}$ x *ML5*$^{tg}$ | 0.0024 | ** |
| *Pten*$^{f/f}$ x mb1-cre x *MD4*$^{tg}$ | *Pten*$^{f/f}$ x mb1-cre x *MD4*$^{tg}$ x *ML5*$^{tg}$ | > 0.9999 | n. s. |
| **Dilution 1:243 (row 6)** | | | |
| *Pten*$^{f/f}$ x *MD4*$^{tg}$ | *Pten*$^{f/f}$ x *MD4*$^{tg}$ x *ML5*$^{tg}$ | 0.0014 | ** |
| *Pten*$^{f/f}$ x mb1-cre x *MD4*$^{tg}$ | *Pten*$^{f/f}$ x mb1-cre x *MD4*$^{tg}$ x *ML5*$^{tg}$ | > 0.9999 | n. s. |

| | | | |
|---|---|---|---|
| **Dilution 1:729 (row 7)** | | | |
| $Pten^{f/f}$ x $MD4^{tg}$ | $Pten^{f/f}$ x $MD4^{tg}$ x $ML5^{tg}$ | 0.0006 | *** |
| $Pten^{f/f}$ x mb1-cre x $MD4^{tg}$ | $Pten^{f/f}$ x mb1-cre x $MD4^{tg}$ x $ML5^{tg}$ | > 0.9999 | n. s. |
| **Dilution 1:2187 (row 8)** | | | |
| $Pten^{f/f}$ x $MD4^{tg}$ | $Pten^{f/f}$ x $MD4^{tg}$ x $ML5^{tg}$ | 0.0005 | *** |
| $Pten^{f/f}$ x mb1-cre x $MD4^{tg}$ | $Pten^{f/f}$ x mb1-cre x $MD4^{tg}$ x $ML5^{tg}$ | > 0.9999 | n. s. |

## Appendix Table S5: related to Fig. 6A

| Kruskal-Wallis test: Dunn's multiple comparisons test | | Adjusted p value | |
|---|---|---|---|
| **IgM MFI** | | | |
| $Pten^{f/f}$ | $Pten^{f/f}$ x $MD4^{tg}$ | 0.0002 | *** |
| $Pten^{f/f}$ x mb1-cre | $Pten^{f/f}$ x mb1-cre x $MD4^{tg}$ | < 0.0001 | **** |
| $Pten^{f/f}$ x $MD4^{tg}$ | $Pten^{f/f}$ x $MD4^{tg}$ x $ML5^{tg}$ | < 0.0001 | **** |
| $Pten^{f/f}$ x $MD4^{tg}$ | $Pten^{f/f}$ x mb1-cre x $MD4^{tg}$ | 0.0627 | n. s. |
| $Pten^{f/f}$ x mb1-cre x $MD4^{tg}$ | $Pten^{f/f}$ x mb1-cre x $MD4^{tg}$ x $ML5^{tg}$ | > 0.9999 | n. s. |
| **IgD MFI** | | | |
| $Pten^{f/f}$ | $Pten^{f/f}$ x $MD4^{tg}$ | 0.1001 | n. s. |
| $Pten^{f/f}$ x mb1-cre | $Pten^{f/f}$ x mb1-cre x $MD4^{tg}$ | 0.2980 | n. s. |
| $Pten^{f/f}$ x $MD4^{tg}$ | $Pten^{f/f}$ x $MD4^{tg}$ x $ML5^{tg}$ | > 0.9999 | n. s. |
| $Pten^{f/f}$ x $MD4^{tg}$ | $Pten^{f/f}$ x mb1-cre x $MD4^{tg}$ | 0.0032 | ** |
| $Pten^{f/f}$ x mb1-cre x $MD4^{tg}$ | $Pten^{f/f}$ x mb1-cre x $MD4^{tg}$ x $ML5^{tg}$ | > 0.9999 | n. s. |

## Appendix Table S6: related to Fig. 6C

| Mann-Whitney U-test: | | P value | |
|---|---|---|---|
| **cHEL** | | | |
| $Pten^{f/f}$ x $MD4^{tg}$ | $Pten^{f/f}$ x $MD4^{tg}$ x $ML5^{tg}$ | 0.2468 | n. s. |
| $Pten^{f/f}$ x $MD4^{tg}$ | $Pten^{f/f}$ x mb1-cre x $MD4^{tg}$ | 0.5368 | n. s. |
| $Pten^{f/f}$ x $MD4^{tg}$ x $ML5^{tg}$ | $Pten^{f/f}$ x mb1-cre x $MD4^{tg}$ x $ML5^{tg}$ | 0.9307 | n. s. |
| $Pten^{f/f}$ x mb1-cre x $MD4^{tg}$ | $Pten^{f/f}$ x mb1-cre x $MD4^{tg}$ x $ML5^{tg}$ | 0.1255 | n. s. |
| **sHEL** | | | |
| $Pten^{f/f}$ x $MD4^{tg}$ | $Pten^{f/f}$ x $MD4^{tg}$ x $ML5^{tg}$ | 0.0043 | ** |
| $Pten^{f/f}$ x $MD4^{tg}$ | $Pten^{f/f}$ x mb1-cre x $MD4^{tg}$ | 0.0087 | ** |
| $Pten^{f/f}$ x $MD4^{tg}$ x $ML5^{tg}$ | $Pten^{f/f}$ x mb1-cre x $MD4^{tg}$ x $ML5^{tg}$ | 0.0087 | ** |
| $Pten^{f/f}$ x mb1-cre x $MD4^{tg}$ | $Pten^{f/f}$ x mb1-cre x $MD4^{tg}$ x $ML5^{tg}$ | 0.2468 | n. s. |
| **Anti-Kappa** | | | |
| $Pten^{f/f}$ x $MD4^{tg}$ | $Pten^{f/f}$ x $MD4^{tg}$ x $ML5^{tg}$ | 0.2468 | n. s. |
| $Pten^{f/f}$ x $MD4^{tg}$ | $Pten^{f/f}$ x mb1-cre x $MD4^{tg}$ | 0.5368 | n. s. |
| $Pten^{f/f}$ x $MD4^{tg}$ x $ML5^{tg}$ | $Pten^{f/f}$ x mb1-cre x $MD4^{tg}$ x $ML5^{tg}$ | 0.6623 | n. s. |
| $Pten^{f/f}$ x mb1-cre x $MD4^{tg}$ | $Pten^{f/f}$ x mb1-cre x $MD4^{tg}$ x $ML5^{tg}$ | 0.5368 | n. s. |

**Appendix Table S7: related to Fig. 7G**

| Mann-Whitney U-test: | | P value | |
|---|---|---|---|
| **WT: PBS vs. TNP-Ova** | | | |
| 10 µg/ml (row 1) | | 0.0095 | ** |
| Dilution 1:3 (row 2) | | 0.0095 | ** |
| Dilution 1:9 (row 3) | | 0.0095 | ** |
| Dilution 1:27 (row 4) | | 0.0095 | ** |
| Dilution 1:81 (row 5) | | 0.0095 | ** |
| Dilution 1:243 (row 6) | | 0.0095 | ** |
| Dilution 1:729 (row 7) | | 0.0143 | * |
| Dilution 1:2187 (row 8) | | 0.1333 | n. s. |
| **IgD$^{-/-}$: PBS vs. TNP-Ova** | | | |
| 10 µg/ml (row 1) | | 0.1017 | n. s. |
| Dilution 1:3 (row 2) | | 0.1212 | n. s. |
| Dilution 1:9 (row 3) | | 0.0931 | n. s. |
| Dilution 1:27 (row 4) | | 0.1320 | n. s. |
| Dilution 1:81 (row 5) | | 0.0281 | * |
| Dilution 1:243 (row 6) | | 0.1429 | n. s. |
| Dilution 1:729 (row 7) | | 0.5000 | n. s. |
| Dilution 1:2187 (row 8) | | 0.1786 | n. s. |
| **Overlay: WT vs. IgD$^{-/-}$** | | | |
| 10 µg/ml (row 1) | | 0.0022 | ** |
| Dilution 1:3 (row 2) | | 0.0022 | ** |
| Dilution 1:9 (row 3) | | 0.0022 | ** |
| Dilution 1:27 (row 4) | | 0.0411 | * |
| Dilution 1:81 (row 5) | | 0.1320 | n. s. |
| Dilution 1:243 (row 6) | | 0.1320 | n. s. |
| Dilution 1:729 (row 7) | | 0.1234 | n. s. |
| Dilution 1:2187 (row 8) | | 0.1905 | n. s. |

## Supplemental Experimental Procedures

**Appendix Table S8 |** Antibodies used in flow cytometric analyses (related to Experimental Procedures)

| Antigen | Species | Conjugate | Clone | Company |
|---|---|---|---|---|
| CD19 | mouse | PerCP-Cy5.5 | 1D3 | BD |
| CD21/CD35 | mouse | APC or PE-Cy7 | 7E9 | BioLegend |
| CD23 | mouse | PE or Biotin | B3B4 | BD |
| CD38 | mouse | APC-Cy7 | 90 | BioLegend |
| CD43 | mouse | FITC | S7 | BD |
| CD45R (B220) | mouse | PE-Cy7 | RA3-642 | eBioscience |
| CD45R (B220) | mouse | BV510 | RA3-6B2 | BioLegend |
| CD90.2 | mouse | PE | 53-2,1 | BD |
| CD95 | mouse | PE-Cy7 | Jo2 | BioLegend |
| GL-7 | mouse | PerCP-Cy5.5 | GL7 | BioLegend |
| IgD | human | APC | IA6-2 | BioLegend |
| IgD | mouse | FITC or Biotin | 11-26 | SouthernBiotech |
| IgD | mouse | APC | 11-26 | eBioscience |
| IgM | human | Alexa Fluor 647 | polyclonal | Jackson |
| IgM | mouse | FITC | polyclonal | SouthernBiotech |
| IgM | mouse | eFluor 450 | eB121-15-F9 | eBioscience |
| IgM | mouse | PE-Cy7 | 2/41 | eBioscience |
| κLC | mouse | APC-Cy7 | 187.1 | BD |
| κLC | mouse | PE | polyclonal | SouthernBiotech |
| κLC | human | Biotin | polyclonal | SouthernBiotech |
| λLC | human | Biotin | polyclonal | SouthernBiotech |

**Appendix Table S9 |** Antibodies used for immunohistochemistry

(related to Experimental Procedures)

| Antigen | Conjugate | Clone | Company |
|---|---|---|---|
| CD169 | FITC | MOMA-1 | AbD serotec |
| CD5 | PE | 53-7.3 | eBioscience |
| IgM | Cy5 | polyclonal | Jackson Immunoresearch |
| PNA | Cy3 | - | vector laboratories |

**Appendix Table S10 |** Primers used for analysis of *LC* gene recombination

(related to Experimental Procedures)

| Primers | Sequence 5'→ 3' |
|---|---|
| *5' Vκ* | ggc tgc ags ttc agt ggc agt ggr tcw ggr ac |
| *3' of Jκ5* | atg cga cgt caa ctg ata atg agc cct ct cc |
| *Srp20-F* | gat tac cgc agg agg agt |
| *Srp20-R* | ag aac gga tga ttg gga a |

**Appendix Table S11 |** TaqMan-probe mixes used for qPCR analyses (related to Experimental Procedures)

TaqMan-probe mixes (Applied Biosystems)

| | |
|---|---|
| *Ighm* | Mm01718957_g1 |
| *Ighd* | Mm03979980_s1 |
| *Cstf64* | Mm00500041_m1 |
| *Ell2* | Mm00507237_m1 |
| *Zfp318* | Mm01273666_m1 |
| *Fcer2* | Mm00442792_m1 |
| *CD79b* | Mm00434143_m1 |
| *Gapdh* | Mm99999915_g1 |

**Chromatin Immunoprecipitation (ChIP)**

Mature B cells were purified from spleens of control mice. Proteins and DNA were crosslinked in 0.75% formaldehyde (Roth). Lysates were prepared and subjected to sonication by using a Bioruptor (Diagenode). DNA fragmentation (100 – 700 bp) was verified by gel electrophoresis. Lysates were pre-cleared with protein G dynabeads (invitrogen) and half of the volume was immunoprecipitated with $\alpha$-FoxO1-antibody (C29H4; Cell Signaling). Precipitated protein-DNA complexes were isolated with protein G dynabeads, decrosslinked and enriched DNA was further purified by using the QIAquick PCR purification kit (Qiagen).

Quantitative PCR was performed by using Maxima SYBR qPCR Master Mix (Thermo Fisher Scientific) and combinations of the primers listed in Appendix Tables S12-14. Specificity of the primer pairs was previously verified by PCR on unfragmented genomic DNA.

**Appendix Table S12 |** Primers used for qPCR amplification of sequences with potential FoxO1-binding sites in the *Zfp318* gene

(related to Experimental Procedures)

| Binding site | Sequence 5'→ 3' for | Sequence 5'→ 3' rev |
|---|---|---|
| *Zfp318-1/2* | cac ttc ctg tga gct gat gga tt | cca cct cac tgt tga ccc aaa |
| *Zfp318-3* | cag ggg cta cct gta agg tct ta | cat gct cag aga ggc tca tgt |
| *Zfp318-4* | gct tat gac att tat aaa taa gca aat cgg ctt | ggc att ggt ggc aca aac ttt |
| *Zfp318-5* | ccc tta ccg tga tag aca aca at | ccg ctc tgg tgt gct aac aca |
| *Zfp318-6* | ggc aaa cac tat ggc att gag cta t | cgc atg tta gat tgt att ttc cca ttg |
| *Zfp318-7* | ggg ctg cta gtc ctg aca aa | cac agc acc cat atg gtg gtt |
| *Zfp318-8* | ggg ctg ata ata cat ggc ttt tct | gag ttt tct agg cca gct agg aat |
| *Zfp318-9* | gcc cac agc gtt ctg gga ta | cct ccc gag cta cac agt aa |
| *Zfp318-10* | ccg aaa ctt ctc cca agc taa taa t | cac ctc cat cag aag tgg atc ttt |
| *Zfp318-11* | tgc cta ggt ggt ccc agt t | ggg tct ggt gga tgt tgt tg |
| *Zfp318 -3.5 kb ctrl* | cca gta tcc aga tac ctt gac cca tat | gag ggc aat tgt gca cta tga at |

**Appendix Table S13 |** Primers used for qPCR amplification of sequences with potential FoxO1-binding sites in the *Ell2* gene

(related to Experimental Procedures)

| Binding site | Sequence 5'→ 3' for | Sequence 5'→ 3' rev |
|---|---|---|
| *Ell2-1* | atg gtg gga cag gac tgt aat | ggg gag ggg ata gtg gat ttt |
| *Ell2-2* | gga gat ttc taa gtg cct gtg tga aa | agc aag cac gtg gca gaa a |
| *Ell2-3* | cag agt gcc tat ccc tgc ttt | cgc ctg ggc ttc aaa cca a |
| *Ell2-4* | cat gac cac cac tca aaa cac att | agg ctg tct caa gag agc tttt |
| *Ell2-5* | ggt tca ccg aga gtt agg aat ata taa gc | gca tcc cat gcc cat tta caa t |
| *Ell2-6/7* | agg agc cac agt tac agc taa | gac aag gga aaa tac cac ttt ttc aaa cat |
| *Ell2-8* | cat gga tgg ggt cct act aga aat | tgc tca cca ggg ttc tag gat t |
| *Ell2-9* | gtg tgg aaa atg ctt agg gtt t | agg act acc ata atc gtg cag act ta |
| *Ell2-10* | gca agc aca cac cca caa | cca cca tac cca gtt gag aac taa ac |
| *Ell2-11* | cca cct cgg agt tta gaa cca aa | ccc agc agg aaa gga tca agt |
| *Ell2-12* | agt act gtt ttg ccc atg caa at | cca aag ggt cag act aag gag att |
| *Ell2-13* | acc aag gta gca gga gag aag t | cac gtg cgc ata gta gga tt |
| *Ell2-14* | tcc ctg gaa gct gtg tgt taa g | gta gtg gta ctg agt agg acg ttt |
| *Ell2-15* | gtg ctg cca gtt gaa aag g | ctg cca caa ggc ctc agt act aa |
| *Ell2-16* | cac agt ggc tgc ctg tag aa | cct ata tgc aag ttg cga cca atg |
| *Ell2-17* | gga ggt gag agc cta gtg cat aa | tgg gct gat tac tga cgt cag a |
| *Ell2 -3.5 kb ctrl* | caa tga cct tgt ctg gcc tct at | gac tct cta gtg agg ggc tgt aa |

**Appendix Table S14 |** Primers used for qPCR amplification of sequences with potential FoxO1-binding sites in the *Fcer2* gene

(related to Experimental Procedures)

| Binding site | Sequence 5'→ 3' for | Sequence 5'→ 3' rev |
|---|---|---|
| *Fcer2-1* | gtg tga cga tgt gtc tgg ctt a | agg gct cac aca gaa gca tat |
| *Fcer2-2* | gac agg tga cca gag gca aa | gca gga ggt cct gac ttg tat aag |
| *Fcer2-3* | act ggg caa agc tga gca ta | acc cat ggg aaa tgg cac tat t |
| *Fcer2-4* | ggc agg ccc att aga ata aca t | ggg tgc agt taa ctt ctt cca ac |
| *Fcer2-5* | gcc aca acc cca tag cat t | cac tgg gcc tgg gtt tt |
| *Fcer2-6* | gga agg cgg ctc agt agg taa a | gtg ctt acc cac tga acc ttg t |
| *Fcer2-7* | gga tcc aaa gcc tgg gga gat a | acc aga ccc gtt gtt aca gat |
| *Fcer2 -3.5 kb ctrl* | ggt aca gtg cac tcc gag cat a | cca tgt ggg tgc tgg aat |
