# Supplementary material for: Pten controls B‐cell responsiveness and germinal center reaction by regulating the expression of IgD BCR
Source: EMBO J. 2019 Apr 23;38(11):e100249. doi: 10.15252/embj.2018100249 (PMC6545559; doi:10.15252/embj.2018100249)
Supplement: Supplementary file 2 — Expanded View Figures PDF [file EMBJ-38-e100249-s002.pdf]

Expanded View Figures

Figure EV1. Pten is required for differentiation of Fo.B cells (related to Figs 2 and 3).

A Serum IgM levels in Pten-deficient *MD4*-transgenic mice, compared to data from $Pten^{f/f}$ and $Pten^{f/f}$ × mb1-cre controls, already shown in Fig 2C. Horizontal lines represent the mean ± SD, and symbols indicate serum IgM concentrations from individual mice (*n*). Statistical significance was calculated by using the Kruskal–Wallis test, n.s. = not significant, $^{*}P \leq 0.05$.

B Mice of the indicated genotypes were sacrificed, and splenic B cells (pre-gated by B220 and CD19 expression) were analyzed for surface expression of CD21 and CD23. Shown data are representative of 11–35 individual mice per genotype.

 © 2019 The Authors

 © 2019 The Authors

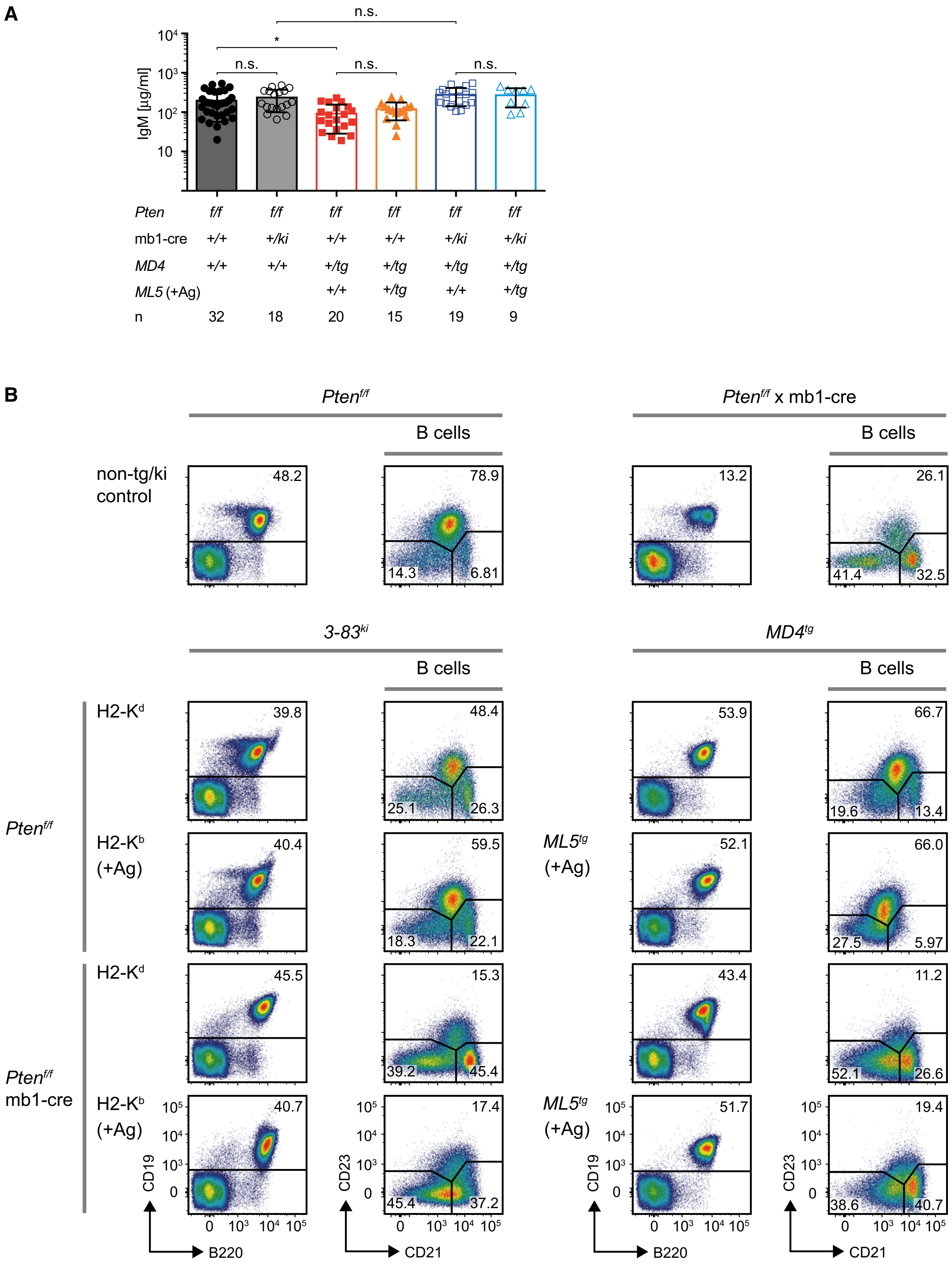
A
n.s.
*
n.s.
n.s.
n.s.
IgM [µg/ml]
Pten f/f f/f f/f f/f f/f f/f
mb1-cre +/+ +/ki +/+ +/+ +/ki +/ki
MD4 +/+ +/+ +/tg +/tg +/tg +/tg
ML5 (+Ag) +/+ +/tg +/+ +/tg
n 32 18 20 15 19 9
B
Pten f/f
Pten f/f x mb1-cre
B cells
non-tg/ki control
3-83 ki
MD4 tg
H2-Kd
H2-Kb (+Ag)
ML5 tg (+Ag)
Pten f/f mb1-cre
CD19
B220
CD23
CD21
48.2
78.9
14.3
6.81
13.2
26.1
41.4
32.5
39.8
48.4
25.1
26.3
53.9
66.7
19.6
13.4
40.4
59.5
18.3
22.1
52.1
66.0
27.5
5.97
45.5
15.3
39.2
45.4
43.4
11.2
52.1
26.6
40.7
17.4
45.4
37.2
51.7
19.4
38.6
40.7

Figure EV1.

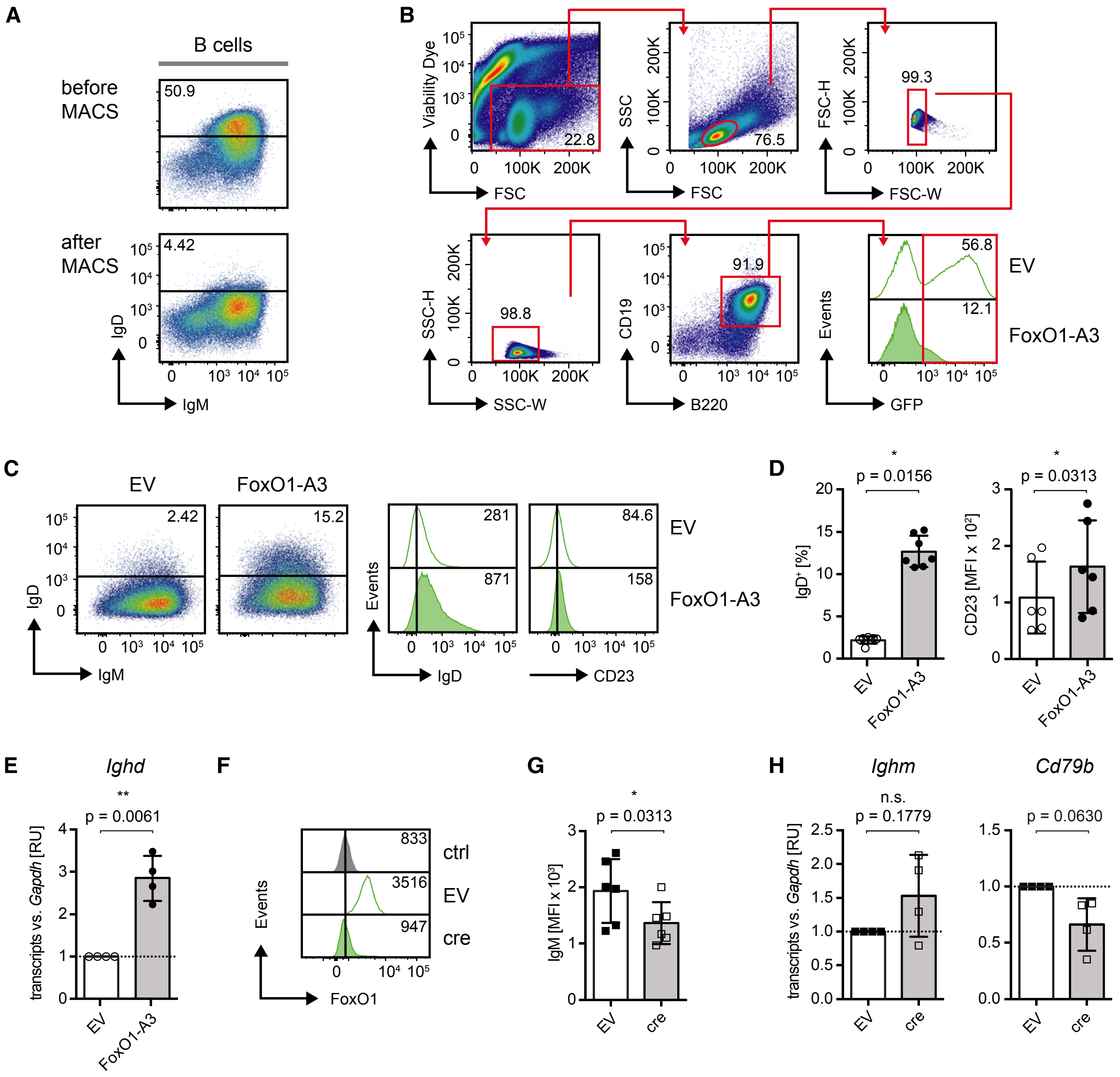

Figure EV2.

 © 2019 The Authors

 © 2019 The Authors

Figure EV2. Pten activates IgD expression and development of Fo.B cells (related to Fig 4).

A Splenic B-1 B cells were isolated, and IgD^{-} cells were purified by MACS-based-negative selection. The enrichment efficiency was assessed by flow cytometric analysis of IgM/IgD surface expression.

B, C Enriched B-1 B cells shown in Fig EV2A were cultured in the presence of 2.5 μg/ml LPS for 1.5 days and subjected to retroviral transduction with expression vectors encoding the constitutively active FoxO1 (FoxO1-A3) or the empty vector (EV). Three days following transduction, cells were gated as shown in (B) and the surface expression of IgM/IgD (dot plots, left panel), IgD, and CD23 (histograms, right panel, numbers indicate the MFI) was measured by flow cytometry and compared between EV- and FoxO1-A3-transduced cells (C).

D Quantified percentages of IgD^{+} cells (left; $n = 7$) and MFI of CD23 (right, $n = 6$), mean ± SD. Symbols indicate data from individual mice. Statistical significance was calculated by using the Wilcoxon matched-pairs signed rank test.

E *Ighd* (Ig-δHC) expression in cells from Fig EV2C, measured by qRT–PCR, mean ± SD. Symbols indicate expression in individual mice ($n = 4$). Statistical significance was calculated by using the one-sample two-tailed *t*-test.

F Expression vectors encoding cre or the EV were introduced into *FoxO1*$^{f/f}$-derived mature splenic B cells, respectively. Three days upon transduction, intracellular FoxO1 expression was measured by flow cytometry and compared between EV- and cre-transduced cells. Staining only with the secondary antibody served as control (ctrl).

G Quantified MFI of IgM ($n = 6$) in cells from Fig 4E, mean ± SD. Symbols indicate data from individual mice. Statistical significance was calculated by using the paired Wilcoxon test.

H *Ighm* (Ig-μHC) and *Cd79b* (Ig-β) expression levels measured at 3 days posttransduction by qRT–PCR in cre- and EV-transduced *FoxO1*$^{f/f}$-derived mature splenic B cells, mean ± SD. Symbols indicate the expression in individual mice ($n = 4$). Statistical significance was calculated by using the one-sample two-tailed *t*-test.

© 2019 The Authors

© 2019 The Authors

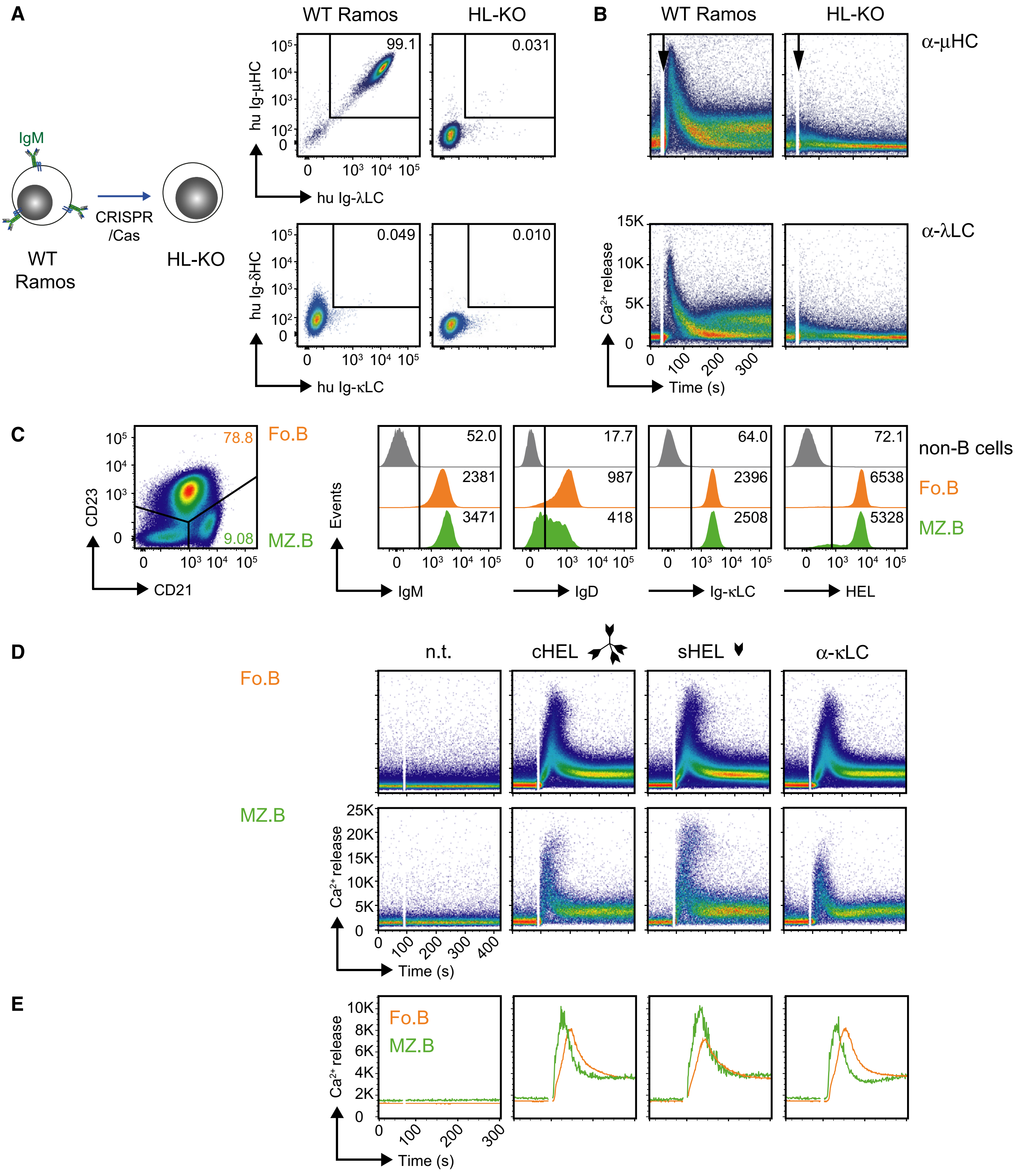

Figure EV3.

© 2019 The Authors

Figure EV3. Establishment of BCR-deficient Ramos cells and Ca^{2+} response in Fo.B and MZ.B cells (related to Fig 5).

A Establishment of BCR-deficient Ramos cells: Schematic overview of BCR *HC* and *LC* gene deletion in Ramos cells by CRISPR/Cas (left). Flow cytometric analysis of BCR surface expression in WT and HC/LC-deficient (HL-KO) Ramos cells (right).

B Comparison of intracellular Ca^{2+} influx measured in WT and HL-KO Ramos cells upon stimulation with 10 μg/ml of α-human μHC (top) or λLC (bottom) antibody, respectively.

C Representative surface expression of IgM, IgD, Ig-κLC, and HEL-binding in splenic $CD21^{lo}/CD23^{+}$ (follicular (Fo.B), orange) and $CD21^{hi}/CD23^{lo/-}$ (marginal zone (MZ.B), green) B cells from *MD4*^{tg} mice, as compared to non-B cells (gray).

D Representative intracellular Ca^{2+} influx measured in $CD21^{hi}/CD23^{lo/-}$ (MZ.B) and $CD21^{lo}/CD23^{+}$ (Fo.B) B cells. Cells were purified by MACS (negative selection), then stained for CD21 and CD23, and subjected to Ca^{2+} measurement upon stimulation with either multivalent HEL (complex cHEL), monovalent (soluble sHEL; both at a concentration of 1 μg/ml), or 10 μg/ml α-mouse κLC antibody, respectively. n.t. = non-treated. Data are representative of three individual mice.

E Overlaid MFI of the calcium influx kinetics displayed in the plots from Fig EV3D: green MZ.B; orange Fo.B.

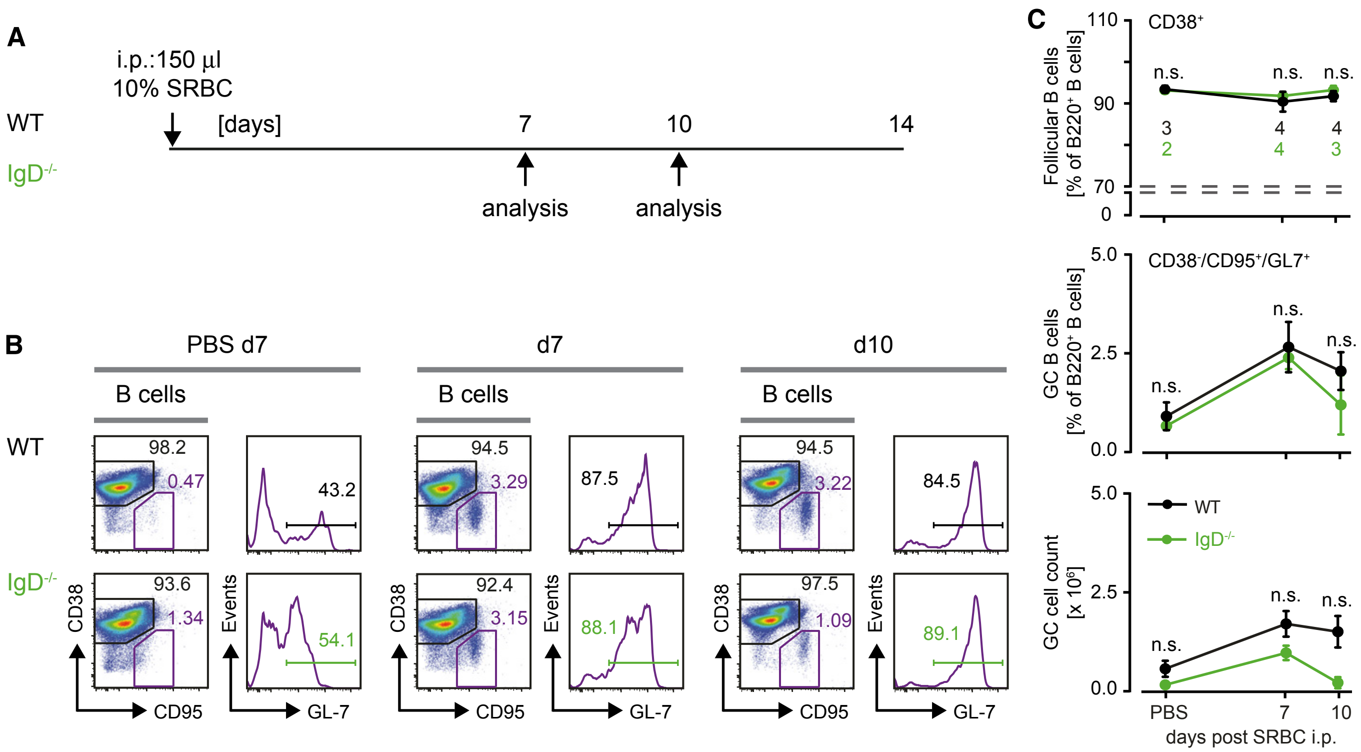

Figure EV4. Immunization of WT and $IgD^{-/-}$ with sheep red blood cells (SRBC) (related to Fig 7).

A Schematic overview of immunization procedure of mice with sheep red blood cells (SRBC) and analysis of germinal center (GC) reactions at different time intervals. Control animals were injected intraperitoneally (i.p.) with PBS and quantified as 0 days of immunization.

B Analysis of GC B cells in spleens from SRBC-immunized WT or $IgD^{-/-}$ mice. Percentages of follicular B cells (Fo.B; $CD38^{+}/CD95^{-}$) and GC B cells ($CD38^{-}/CD95^{+}$) are depicted in the plots. GC B cells ($CD38^{-}/CD95^{+}$) were further analyzed for GL-7 expression.

C Quantification of follicular B cells (top, percentages) and $CD38^{-}/CD95^{+}/GL\text{-}7^{+}$ GC cells (percentages and absolute numbers, bottom panels) in WT (black) and $IgD^{-/-}$ (green) animals after 7 and 10 days postimmunization, mean ± SD (number of animals indicated in the top plot). Statistical significance was analyzed by a two-tailed unpaired *t*-test, n.s. = not significant.
